# MEG Frequency Analysis Depicts the Impaired Neurophysiological Condition of Ischemic Brain

**DOI:** 10.1371/journal.pone.0168588

**Published:** 2016-12-16

**Authors:** Shinichi Sakamoto, Hidetoshi Ikeda, Naohiro Tsuyuguchi, Takehiro Uda, Eiichi Okumura, Takashi Asakawa, Yasuhiro Haruta, Hideki Nishiyama, Toyoji Okada, Hajime Kamada, Kenji Ohata, Yukio Miki

**Affiliations:** 1 Department of Diagnostic and Interventional Radiology, Osaka City University Graduate School of Medicine, Osaka, Japan; 2 Department of Neurosurgery, Osaka City University Graduate School of Medicine, Osaka, Japan; 3 Medical Imaging Business Department, Ricoh Company, Ltd., Kanazawa, Japan; 4 Applied Electronics Laboratory, Kanazawa Institute of Technology, Kanazawa, Japan; 5 Nihon Medi-Physics Corporation, Tokyo, Japan; 6 Department of Clinical Laboratory, Hokuto Hospital, Obihiro, Japan; 7 Department of Neurosurgery, Hokuto Hospital, Obihiro, Japan; University of North Carolina at Chapel Hill, UNITED STATES

## Abstract

**Purpose:**

Quantitative imaging of neuromagnetic fields based on automated region of interest (ROI) setting was analyzed to determine the characteristics of cerebral neural activity in ischemic areas.

**Methods:**

Magnetoencephalography (MEG) was used to evaluate spontaneous neuromagnetic fields in the ischemic areas of 37 patients with unilateral internal carotid artery (ICA) occlusive disease. Voxel-based time-averaged intensity of slow waves was obtained in two frequency bands (0.3–4 Hz and 4–8 Hz) using standardized low-resolution brain electromagnetic tomography (sLORETA) modified for a quantifiable method (sLORETA-qm). ROIs were automatically applied to the anterior cerebral artery (ACA), anterior middle cerebral artery (MCAa), posterior middle cerebral artery (MCAp), and posterior cerebral artery (PCA) using statistical parametric mapping (SPM). Positron emission tomography with ^15^O-gas inhalation (^15^O-PET) was also performed to evaluate cerebral blood flow (CBF) and oxygen extraction fraction (OEF). Statistical analyses were performed using laterality index of MEG and ^15^O-PET in each ROI with respect to distribution and intensity.

**Results:**

MEG revealed statistically significant laterality in affected MCA regions, including 4–8 Hz waves in MCAa, and 0.3–4 Hz and 4–8 Hz waves in MCAp (95% confidence interval: 0.020–0.190, 0.030–0.207, and 0.034–0.213), respectively. We found that 0.3–4 Hz waves in MCAp were highly correlated with CBF in MCAa and MCAp (*r = 0*.*74*, *r = 0*.*68*, respectively), whereas 4–8 Hz waves were moderately correlated with CBF in both the MCAa and MCAp (*r = 0*.*60*, *r = 0*.*63*, respectively). We also found that 4–8 Hz waves in MCAp were statistically significant for misery perfusion identified on ^15^O-PET (*p<0*.*05*).

**Conclusions:**

Quantitatively imaged spontaneous neuromagnetic fields using the automated ROI setting enabled clear depiction of cerebral ischemic areas. Frequency analysis may reveal unique neural activity that is distributed in the impaired vascular metabolic territory, in which the cerebral infarction has not yet been completed.

## Introduction

Imaging of the ischemic penumbra [1, 2], in which cerebral blood flow is reduced but neurons have not yet become necrotic, is currently a key area of interest in the investigation of cerebrovascular disease [3, 4] because ischemic penumbra, which is evident in the brain of patients suffering from ischemic cerebrovascular disease, commonly progresses to cerebral infarction [5]. Accurate identification of ischemic penumbra prior to intervention, including vascular reconstructive surgery and endovascular treatment, may prevent the development of cerebral infarction [6, 7].

Ischemic penumbra can be assessed on the basis of the clinical symptoms (e.g., transient ischemic attack, TIA), anatomical findings (computed tomography, CT; and magnetic resonance imaging, MRI), and by measuring cerebral blood flow and metabolism (single photon emission computed tomography (SPECT) and positron emission tomography (PET)). Diffusion-weighted imaging (DWI) and perfusion-weighted imaging (PWI) of MRI have been utilized to evaluate ischemic cerebrovascular disorders, and the area of DWI-PWI mismatch is thought to represent the area of reversible ischemia [8, 9]. CT perfusion imaging has also been utilized to evaluate the ischemic area in the acute stage of stroke [10, 11]. However, CT and MRI cannot always determine the area of reversible ischemia because these methods image the secondary change of tissues caused by a decrease in cerebral blood flow. Although PET and SPECT are the gold standards for evaluating cerebral ischemia, they cannot be casually performed due to risks such as radiation exposure or arterial blood sampling.

Electroencephalography (EEG), which is a method of electrophysiological examination, can also demonstrate the area of reversible cerebral ischemia (ischemic penumbra) as slow waves [12–14]. However, in the age of MRI, EEG has minimal clinical applications for evaluating cerebral ischemia because it has markedly low spatial resolution, which is due to the presence of the scalp, skull, and cerebrospinal fluid between the brain and the electrodes. In contrast, magnetoencephalography (MEG), which is becoming used more frequently, enables direct capture of cerebral neural activities and solves the problems associated with the low spatial resolution of EEG [15]. Furthermore, MEG is a completely non-invasive imaging modality, which allows repeated examinations without stress to the patient. Previous studies conducted using MEG for assessment of slow-wave distributions have confirmed increased slow-wave intensity around cerebral infarctions [16–21]. However, analysis frequencies for slow waves have varied from delta to theta in these previous studies, and interpretation of the results also differed (Butz et al. [16], 0.5–3 Hz; Kamada et al. [17], 2–6 Hz; Stippich et al. [19], 2–6 Hz; Seki et al. [20], 6–8 Hz; Ohtomo et al. [21], 6–8 Hz).

In a previous study, we presented an imaging technique for slow-wave analysis using adaptive spatial filtering [22] in which the estimated areas of slow-wave distribution were consistent with the areas of hypovascularity revealed by brain SPECT imaging; we also reported the disappearance of slow waves associated with improvement in hypovascularity following vascular reconstructive surgery. We subsequently developed standardized low-resolution brain electromagnetic tomography modified for a quantifiable method (sLORETA-qm), which is a spatial filtering technique that enables quantitative assessment of the current intensity of intracerebral neural activity [23], and reported that sLORETA-qm enables both quantitative imaging of cerebral slow-wave distribution in patients with ischemic cerebrovascular disease, and visualization of the changes in slow-wave distribution that are associated with the cerebrovascular condition [24]. However, these old analysis methods are highly operator dependent because analysts subjectively select channels or subjectively decide the number of sources and source locations, and because the calculated current intensity is expressed as the maximum value of the distribution; accordingly, these methods are not necessarily appropriate for the investigation of patients with ischemic cerebrovascular disease in whom evaluation of the cerebral vascular territory is preferable.

In the present study, slow waves of patients with internal carotid artery (ICA) occlusive disease were acquired with MEG, divided into two frequency bands (delta: 0.3–4 Hz, theta: 4–8 Hz), and quantitatively imaged using sLORETA-qm. Automated regions of interest (ROIs), which were congruent with the cerebral vascular territory, were applied to the estimated source images of MEG (MEG images) for objective evaluation in relation to the distribution and intensity of slow waves. Automated ROI settings in the same manner as for MEG images were also applied to positron emission tomography with ^15^O-gas inhalation (^15^O-PET) images to investigate the correlation between distributions and intensities on MEG and PET images.

Our aims of this study were to elucidate the behavior of slow waves, delta waves, and theta waves in the ischemic brain where only minimal neurological symptoms developed despite a marked decreased in hemodynamic reserve (“misery perfusion”). We also investigated the imaging potential of MEG as a non-invasive imaging modality for predicting the area of misery perfusion “ischemic penumbra”.

## Materials and Methods

### Subjects

Thirty-seven consecutive patients with unilateral carotid artery occlusive disease (males, n = 26; females, n = 12; age range, 35–89 years; mean age, 62.9 years) were enrolled in the present study. The clinical profiles and characteristics of the patients are summarized in Table 1. Fifteen patients were diagnosed with asymptomatic brain disease at a health check service, and 22 patients visited the outpatient department of Hokuto Hospital with the chief complaints of dizziness, dysarthria, TIA, and mild hemiparesis from January 2006 to March 2007. The clinical symptoms on admission were minor (better than grade 2 on the modified Rankin Scale [25]), even in patients with completed stroke. All patients were free of any psychological disease, and none was taking any drugs that affect the neural condition of the cerebrum.

**Table 1:** Patient characteristics.

Characteristic		Patients number (n = 37)
Gender	Male	26
	Female	11
Age	mean (range)	62.8 (35–89)
Diagnosis	ICO	5
	ICS	17
	MCO	5
	MCS	10
Region side	Right	20
	Left	17
Symptom	Hemiparesis	6
	TIA	8
	other	8
	none	15
MRI lesion	cortical and/or subcortical (single)	2
	cortical and/or subcortical (multiple)	6
	cortical and/or subcortical (multiple) + basal ganglia	9
	none	20
DWI high	present	10
	absent	27
Outcome (mRS)	0	28
	1	3
	2	6

ICO: internal carotid artery occlusion; ICS: internal carotid artery stenosis; MCO: middle cerebral artery occlusion; MCS: middle cerebral artery stenosis. TIA: transient ischemic attack mRS: modified Rankin Scale

All patients underwent MRI including magnetic resonance angiography (MRA) to evaluate the ischemic lesions in the cerebrum. Digital subtraction angiography with Seldinger’s method was also performed to evaluate occlusive lesions of the cervical and intracranial arteries. Cervical lesions were defined as those having more than 70% stenosis according to the method used in the North American Symptomatic Carotid Endarterectomy Trial [26]. Intracranial lesions of the ICA and middle cerebral artery (MCA) were also defined as those having more than 70% stenosis. Underlying pathology was unilateral ICA occlusion in five patients, unilateral ICA stenosis in 17 patients, unilateral MCA occlusion in five patients, and unilateral MCA stenosis in 11 patients. None of the patients had any history of severe cerebral infarction on admission. Patients with any other structural lesions such as tumors, vascular malformations, cerebral contusions, or anomalies were excluded from the study.

The present study conformed to the ethical principles of the Helsinki Declaration and was approved by the Ethics Committee of Hokuto Hospital. Written informed consent was obtained from each patient after the aims and procedures of the study had been explained in detail.

### MRI

MRI was performed using a 3.0-T scanner (SIGNA EXCITE 3.0T, GE Healthcare, Milwaukee, WI) with a standard head coil. T1-weighted imaging (T1WI), T2-weighted imaging (T2WI), and fluid attenuated inversion recovery (FLAIR) images were acquired to detect cerebral ischemic lesions. T1WI using a T1-weighted spoiled gradient recalled echo sequence was obtained for co-registration with MEG data. DWI including apparent diffusion coefficient (ADC) mapping was also acquired to differentiate between acute and old infarctions. The cerebral arteries were evaluated using time-of-flight MRA.

### MRI interpretation and representation of an ischemic lesion using statistical parametric mapping

Ischemic lesions were assessed using T1WI, T2WI, and DWI of the whole brain in all patients. Lesions that showed both signal hypointensity on T1WI and signal hyperintensity on T2WI were considered old lesions. Lesions that showed both signal hyperintensity on DWI and a decreasing ADC were considered acute lesions. Ischemic lesions identified on the individual MRIs of all 37 patients were anatomically standardized and presented using statistical parametric mapping (SPM) (SPM8, The Wellcome Trust Centre for Neuroimaging, London, UK).

### PET

PET was performed using an Advance NXi PET Scanner (GE Yokogawa Medical Systems, Tokyo, Japan) by the ^15^O gas inhalation (steady-state) method to measure CBF, cerebral metabolic rate of oxygen (CMRO_2_), oxygen extraction fraction (OEF), and cerebral blood volume (CBV) [27–29]. The methods used in the present study were described in detail previously [30]. CBF and OEF data were anatomically standardized by SPM.

### MEG

MEG was performed using a 160-channel whole-head-type gradiometer (MEG vision PQ1160C; Yokogawa, Kanazawa, Japan) to measure spontaneous cerebral neuromagnetic fields with the patient in the supine position with the eyes closed. While the patient was awake but not moving, and in the absence of environmental noise, 600-s data were recorded under the following conditions: sampling frequency, 500 Hz; band-pass filter, 0.16–200 Hz; and notch filter, 50 Hz. Acquired neuromagnetic fields were divided into two frequency bands: delta, 0.3–4 Hz; and theta, 4–8 Hz. Frequency analysis was then performed for each using sLORETA-qm, which is a previously reported technique [23]. Quantitative images obtained from this analysis were also spatially standardized by SPM.

### sLORETA-qm

sLORETA-qm is a spatial filtering technique for quantifying the cerebral neuromagnetic fields as described in detail previously [23] [24]. Spatial filtering techniques are an alternative for analyzing magnetic brain activities that are difficult to analyze using equivalent current dipole (ECD) models [31]. Standardized low-resolution brain electromagnetic tomography (sLORETA), which is the original form of sLORETA-qm, is a non-adaptive spatial filtering technique, and has no localization bias under ideal conditions [32]. Source reconstruction by sLORETA can be performed without sensor selection, and therefore excludes the arbitrariness of sensor selection. Moreover, sLORETA can also provide pseudo-statistical values that can be used as estimates of activity. These properties are highly advantageous for quantitative analysis of MEG in the clinical setting. To utilize sLORETA as a quantifiable method, sLORETA-qm was developed.

### Processes for quantitative magnetic source imaging using sLORETA-qm

Details of the method of quantitative magnetic source imaging using sLORETA-qm were previously described [24].

First, noise components such as eye movements, head movements, metal artifacts, and other environmental noises were decomposed by principal component analysis and removed based on visual inspection for these spatial patterns and time courses. Time segments containing unremovable noise after principal component analyses were discarded from further processing. Band-pass filtering (delta, 0.3–4 Hz; theta, 4–8 Hz) was applied to measured data, and conventional sLORETA analysis was calculated using an artifact-free period in each band-passed dataset. In this step, the lead field vector and matrix were calculated with a spherical model fit to the brain area of each subject, and the brain area in the spherical model was divided into approximately 5000 voxels with 7 mm/7 mm/7 mm spacing. The actual voxel count depended on the head size of each subject. MEG data from all sensors were used for this calculation, thus avoiding arbitrary sensor selection. Voxels with spatially or locally maximum intensity at each time point were selected and transformed to obtain quantitative values. This operation can be performed automatically by referring to the spherical model of each subject. Calculated source intensity for each voxel was averaged for the selected time interval in which unremovable noise for calculations did not exist, and images of time-averaged sources were superimposed on individual MRI scans.

Based on the above algorithm, quantitative analysis of spontaneous activities was computed with sLORETA-qm using custom software developed using MATLAB version 7.3 (The MathWorks, Natick, MA).

### Automated ROI setting in quantitative imaging of PET and MEG

**Fig 1:**
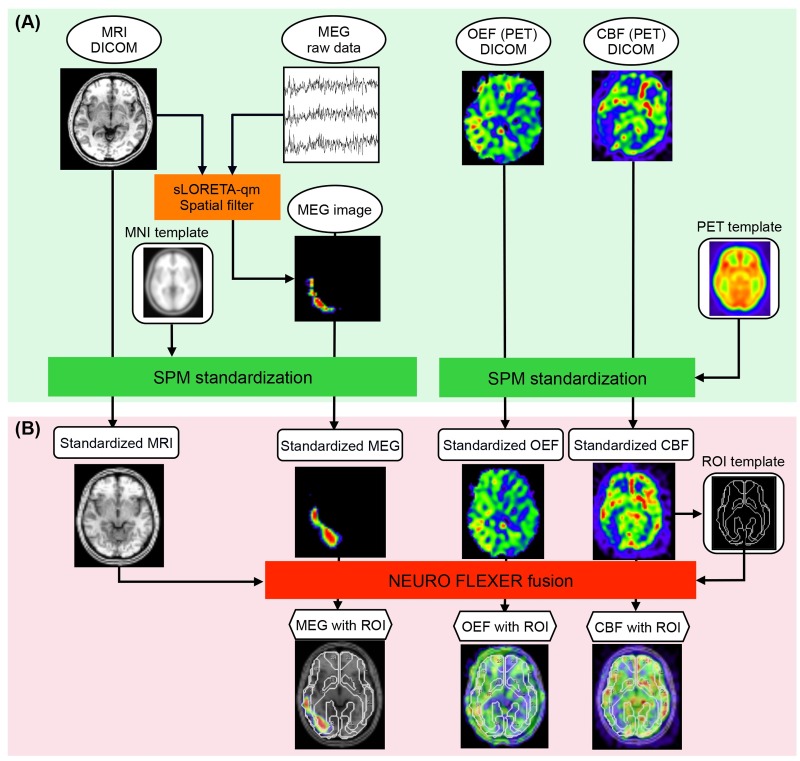
Pipelines for automated ROI setting for quantitative images of PET and MEG. (A) The first step was standardization using SPM. A MEG source image was acquired using a sLORETA-qm spatial filter from the slow spontaneous magnetic fields. MRI-T1WI, CBF in the PET image, and the MEG source image were spatially standardized by SPM using a pre-ordered template of MRI-T1WI and CBF in PET. OEF in the PET image was also standardized by SPM using the template of CBF in PET. (B) The second step was automated ROI constitution using NEUROFLEXER. The pre-ordered ROI template can be applied to the different modalities e.g., MEG and PET, through the normalized MRI.

The flow chart of this process is shown in Fig 1.

MRI-T1WI using a T1-weighted spoiled gradient recalled echo sequence was converted to a standardized image using the pre-ordered Montreal Neurological Institute template (MNI 305) of MRI-T1WI by SPM version 8 (SPM8, The Wellcome Trust Centre for Neuroimaging). MEG was standardized using this conversion matrix of MRI by SPM. CBF in PET images was converted to standardized images using the template of CBF in PET. The conversion matrix was used for standardization of personal OEF in PET images. For all data acquisitions, the same imaging parameters of a matrix size of 64 × 64 were applied.

The automated ROI constitution software ‘NEUROFLEXER’ with the functions of NEUROSTAT developed by Ogura et al. was used for ROI constitution [33]. This software can automatically set a pre-ordered ROI template into the first study (e.g., PET) and can apply the same ROI template to the second study (e.g., MEG) through the normalized MRI. Consequently, any pairs of normalized PET and MEG images can be evaluated in the same ROI in this manner.

**Fig 2:**
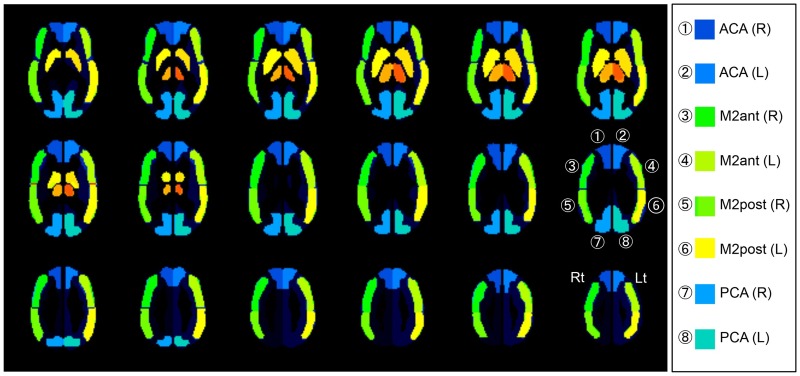
Automated ROI constitution focusing of the cerebral vascular territory using NEUROSTAT. ROIs were set to cover the major vascular regions, assuming patients with cerebral vascular disease. ACA, MCAa, MCAp, and PCA regions were generated by NEUROSTAT [33]. MCAa and MCAp are comparable to M2ant and M2post in this figure, respectively.

We evaluated the slow wave intensity of MEG and CBF, OEF of PET in relation to the ROI template, and the anterior cerebral artery (ACA), anterior part of the MCA (MCAa), posterior part of the MCA (MCAp), and posterior cerebral artery (PCA), which were generated by NEUROSTAT [33] (Fig 2).

### Statistical analysis

Absolute values of CBF, OEF, and MEG were not suitable for statistical analysis including the correlation coefficient, because absolute values had wide variability among patients. Therefore, the laterality index (LI), defined as follows: (value of affected side—value of normal side)/(value of affected side + value of normal side) [34], was employed to simplify the statistical analysis of PET and MEG values (PET-LI, MEG-LI). LIs were imaged in the pair of symmetrical voxels on the affected side and normal side. A positive value of LI on the affected side was consistent with a negative value of LI on the normal side for symmetrical voxels.

We used the 95% confidence interval (95% CI) of the mean LI to assess the statistically significant laterality of PET and MEG in each ROI. The 95% CI of LI was determined using the Bootstrap method [35]. The non-parametric Spearman's rank correlation coefficient was used to analyze the correlation between the absolute value of PET-LI (CBF-LI, OEF-LI) and MEG-LI.

Mann-Whitney U analyses were performed to evaluate the differences in MEG-LI in each ROI between the two groups of ischemic conditions, Group 1 and Group 2, which were defined as follows on the basis of the ^15^O-PET findings: Group 1 was defined as either CBF-LI more than –0.03, or OEF-LI less than 0.01 in the ROI of the MCA; Group 2 was defined as both CBF-LI less than –0.03 and OEF-LI more than 0.01 in the ROI of the MCA. These cut-off levels were determined according to the median and inter-quartile range for CBF-LI of –0.033 and 0.0474, and OEF-LI of 0.00795 and 0.01505, respectively. Thirty patients were classified into Group 1, and seven patients were classified into Group 2. In this classification, cerebral vascular reserve was more severely impaired in Group 2 than in Group 1. The range and mean of the ages of patients in Groups 1 and 2 did not differ significantly (age range, 39 to 89 years; mean age 63.7 years in Group 1; age range, 35 to 76 years; mean age, 59.3 years in Group 2).

Univariate logistic regression analyses were also performed to evaluate the frequency bands and regions of slow waves associated with a severe cerebral ischemic condition. Group 2 (group with severely impaired cerebral vascular reserve) is an objective variable, and MEG-LIs of each frequency band in each ROI are explanatory variables in this statistical analysis. 95% CIs were calculated for the odds ratios.

All statistical analyses were performed with SPSS Statistics 19 (IBM, Chicago, IL). The level of statistical significance was defined as *p* < 0.05 or *p* < 0.01.

## Results

### MRI lesions using SPM

**Fig 3:**
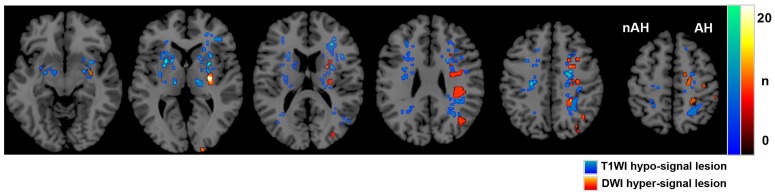
MRI lesion distribution of 37 patients using SPM. This image shows the lesion distribution on MRI of the 37 patients included in the present study. The numbers of patients with ischemic lesions are shown by color-coding. Lesions that were hypointense on T1WI are colored blue to green, whereas lesions that were hyperintense on DWI are colored red to yellow. The lesions are seen predominantly in the affected hemisphere, and the distributions show the watershed type of infarction. The images were anatomically standardized by SPM. AH: affected hemisphere, nAH: non-affected hemisphere.

Fig 3 shows the distribution of lesions in the 37 patients. The images were anatomically standardized by SPM. Infarct lesions were located in the cortical, subcortical, and deep white matter regions bilaterally, but predominantly in the affected hemisphere; lesion distributions presented as the watershed type of infarction. Lesions were also located in the basal ganglia. Acute lesions were located only on the affected side.

### Results of ^15^O-PET and MEG

**Fig 4:**
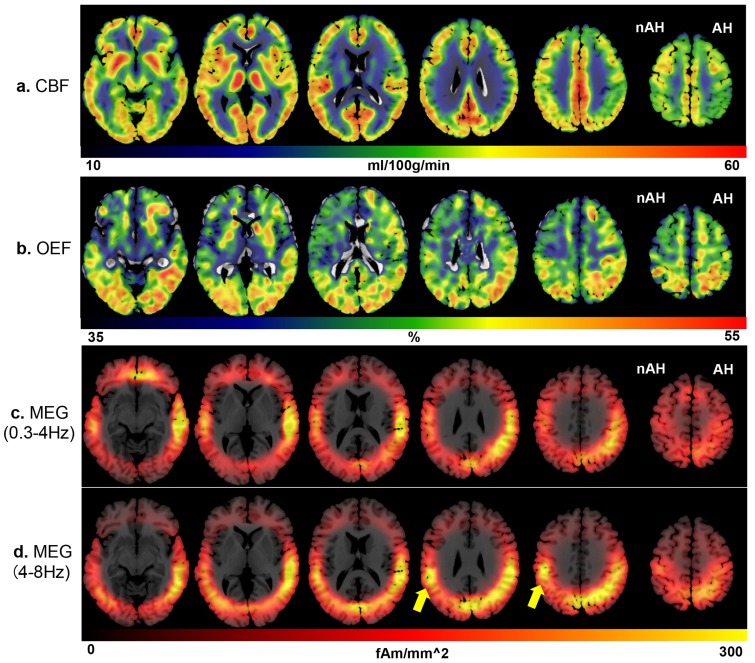
The absolute values from ^15^O-PET and MEG images of the 37 patients using SPM. These images show the absolute value from ^15^O-PET and MEG images of the 37 patients included in the present study, showing CBF (a), OEF (b), 0.3- to 4-Hz waves (c), and 4- to 8-Hz waves (d). The averages of the absolute values are encoded with color (values are shown in the color bars). The images were anatomically standardized by SPM. CBF values are decreased and OEF values are increased predominantly in the affected hemisphere. Both 0.3- to 4-Hz and 4- to 8-Hz waves of MEG are distributed predominantly in the affected hemisphere, from the posterior temporal to parietal regions. The absolute value from MEG images also suggested that the 4- to 8-Hz waves were distributed in the parietal area contralateral to cerebral hemisphere affected by the occlusive ICA lesion (Fig 4d, yellow arrows). AH: affected hemisphere, nAH: non-affected hemisphere.

**Fig 5:**
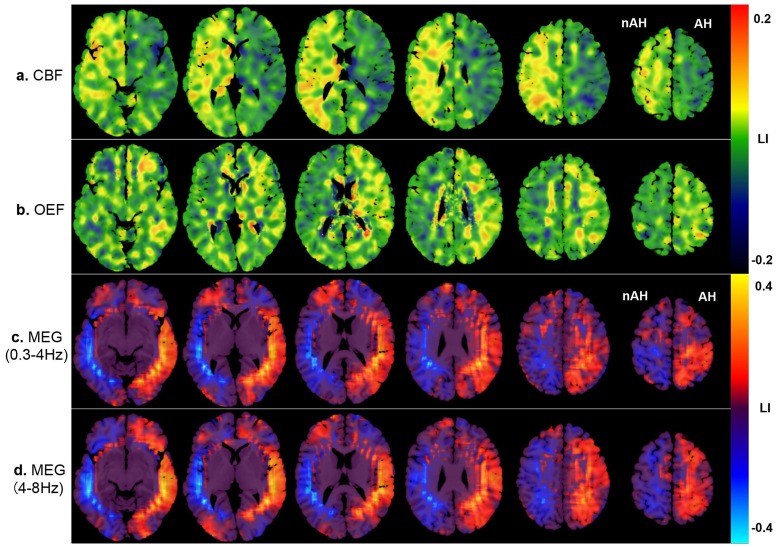
LI from the ^15^O-PET and MEG images of the 37 patients using SPM. These images show the LI of the ^15^O-PET and MEG images of the 37 patients included in the present study, showing CBF (a), OEF (b), 0.3- to 4-Hz waves (c), and 4- to 8-Hz waves (d). The averages of the LI are encoded with color (values are shown in the color bars). The images were anatomically standardized by SPM. CBF values are decreased and OEF values are increased in the affected hemisphere. Both 0.3- to 4-Hz and 4- to 8-Hz MEG waves are distributed in the affected hemisphere, predominantly from the posterior temporal to parietal regions. AH: affected hemisphere, nAH: non-affected hemisphere.

**Fig 6:**
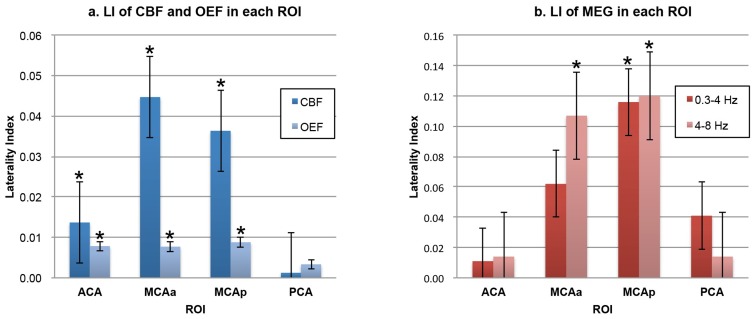
Bar graphs showing the LI of ^15^O-PET and MEG. The ^15^O-PET results showed statistically significant lateralities in CBF and OEF in the ROIs of the ACA, MCAa, and MCAp (a). The MEG results showed statistically significant lateralities for 4- to 8-Hz waves in the MCAa, and for 0.3- to 4-Hz and 4- to 8-Hz waves in the MCAp (b). Data are expressed as the mean ± standard error of the mean (n = 37). Significant laterality was determined with the 95% CI with the Bootstrap method [35]. (*: Range of 95% CI represents a positive value.)

Detailed results of investigation using ^15^O-PET and MEG are shown in Table 2. Fig 4 shows the images of ^15^O-PET and MEG, which are the averages of the absolute values of the 37 patients, and Fig 5 shows the images of ^15^O-PET and MEG, which are the averages of the LI of the 37 patients included in the present study for the CBF (a), OEF (b), 0.3- to 4-Hz waves (c), and 4- to 8-Hz waves (d). The images were anatomically standardized by SPM. The values are encoded with color (shown in the color bars). Fig 6 shows bar graphs of the LI of ^15^O-PET (a) and MEG (b).

**Table 2:** Results of investigation with 15O gas PET and MEG.

Modality		ROI	AH (mean, SD)		nAH (mean, SD)		LI (mean, SD)		95%CI of LI	
15O gas PET	CBF (ml/100g/min)	ACA	35.74	8.44	36.66	8.22	-0.014	0.030	-0.024 − -0.006	*
		MCAa	36.32	8.89	39.55	8.96	-0.045	0.049	-0.062 − -0.031	*
		MCAp	39.50	9.41	42.27	9.26	-0.036	0.042	-0.050 − -0.024	*
		PCA	43.53	10.39	43.48	9.90	-0.001	0.028	-0.010 − 0.009	
	OEF (%)	ACA	43.29	7.52	42.64	7.81	0.008	0.022	0.001 − 0.015	*
		MCAa	42.97	7.63	42.31	7.51	0.008	0.020	0.002 − 0.014	*
		MCAp	46.16	7.61	45.45	7.65	0.008	0.017	0.003 − 0.014	*
		PCA	46.55	7.43	46.36	8.04	0.003	0.032	-0.007 − 0.013	
MEG	0.3–4 Hz (fAm/mm^2)	ACA	86.52	79.77	87.60	80.36	0.011	0.301	-0.087 − 0.106	
		MCAa	108.55	69.04	95.91	52.67	0.062	0.250	-0.018 − 0.138	
		MCAp	170.00	144.88	122.99	84.70	0.116	0.284	0.030 − 0.207	*
		PCA	77.44	50.56	71.59	54.05	0.041	0.143	-0.007 − 0.086	
	4–8 Hz (fAm/mm^2)	ACA	26.88	19.34	25.03	18.01	0.014	0.195	-0.051 − 0.077	
		MCAa	78.29	45.56	62.71	36.15	0.107	0.291	0.020 − 0.190	*
		MCAp	177.01	106.28	142.60	104.81	0.120	0.298	0.034 − 0.213	*
		PCA	106.58	60.61	105.01	63.99	0.014	0.139	-0.031 − 0.061	

ROI: region of interest. ACA: anterior cerebral artery; MCAa: anterior branch of middle cerebral artery; MCAp: posterior branch of middle cerebral artery; PCA: posterior cerebral artery. AH: affected hemisphere; nAH: non-affected hemisphere

*: statistically significant laterality, confidence level at 0.95 by Bootstrap method [35].

In the PET study, CBF in the ROI of the ACA, MCAa, and MCAp showed statistically significant laterality (mean ± SD: –0.014 ± 0.030, –0.045 ± 0.049, and –0.042 ± 0.042; 95% CI: –0.0235 to –0.0058, –0.0616 to –0.0309, and –0.0502 to –0.0242, respectively). OEF in the ROI of the ACA, MCAa, and MCAp showed statistically significant laterality (mean ± SD: 0.008 ± 0.022, 0.008 ± 0.020, and 0.008 ± 0.017; 95% CI: 0.0014 to 0.0148, 0.0015 to 0.0144, and 0.0025 to 0.0141, respectively). Laterality of CBF and OEF in the ROI of the PCA was not statistically significant (Fig 6a). The imaging findings support these statistical analyses (Fig 5a and 5b). These results indicate the presence of decreased CBF and increased OEF in regions ipsilateral to the occlusive lesion of the ICA system. These patients were therefore diagnosed with reduced cerebral blood flow and metabolic reserve (misery perfusion) in the affected cerebral hemisphere, except for the vertebro-basilar circulatory area.

In the MEG study, 0.3- to 4-Hz waves in the ROI of the MCAp and 4- to 8-Hz waves in the ROI of the MCAa and MCAp showed statistically significant laterality (mean ± SD: 0.116 ± 0.284, 0.107 ± 0.291, and 0.120 ± 0.298; 95% CI: 0.0302 to 0.2071, 0.0203 to 0.1902, and 0.0338 to 0.2127, respectively) (Fig 6b). The imaging findings also supported these statistical analyses: slow-wave distributions in both frequency bands analyzed using sLORETA-qm (0.3–4 Hz, 4–8 Hz) were predominantly distributed in the cerebral hemisphere corresponding to the side ipsilateral to the occlusive lesion of the ICA system. Slow-wave distributions were more dominant in the temporo-parietal area at both 0.3–4 Hz and 4–8 Hz (Fig 5c and 5d).

Of note, the absolute value of MEG images also suggested that the 4- to 8-Hz waves were distributed in the parietal area contralateral to the cerebral hemisphere affected by the occlusive ICA lesion (Fig 4d, yellow arrows).

### Correlation coefficient between MEG and each of CBF and OEF in each ROI

Detailed results of the correlation coefficients between the MEG-LI and each of CBF-LI and OEF-LI in each ROI are shown in the S1 Table.

**Fig 7:**
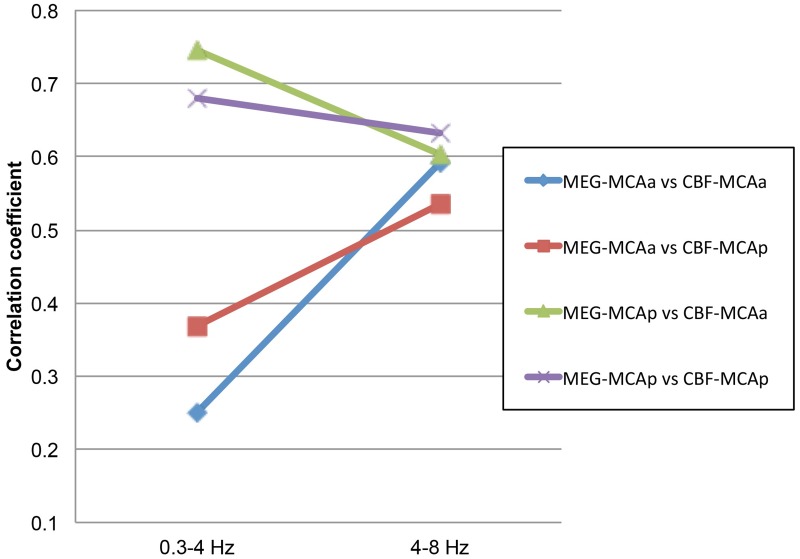
Correlation coefficient between the CBF and MEG in the ROIs of the MCAa and MCAp. Line plot graph showing absolute values of the correlation coefficient between CBF-LI and MEG-LI in the ROIs of the MCAa and MCAp. We observed a high correlation between 0.3- to 4-Hz wave laterality in the MCAp and CBF-LI in the MCAa (*r = 0*.*68*, *p < 0*.*01*) and MCAp (*r = 0*.*74*, *p < 0*.*01*), whereas no significant correlation was recognized between 0.3- to 4-Hz wave laterality in the MCAa and CBF-LI in the MCAa (*r = 0*.*25*, *p = 0*.*13*) or MCAp (*r = 0*.*37*, *p = 0*.*02*). We found a moderate correlation between 4- to 8-Hz wave laterality and CBF-LI in the ROIs of the MCAa and MCAp.

Regarding the absolute value of the correlation coefficient between CBF-LI and MEG-LI, 0.3- to 4-Hz wave laterality in the MCAp was highly correlated with CBF-LI in the MCAa (*r = 0*.*68*, *p < 0*.*01*) and MCAp (*r = 0*.*74*, *p < 0*.*01*), whereas no significant correlations were recognized between 0.3- to 4-Hz wave laterality in the MCAa and CBF-LI in the MCAa (*r = 0*.*25*, *p = 0*.*13*) or MCAp (*r = 0*.*37*, *p = 0*.*02*). Meanwhile, 4- to 8-Hz wave laterality was moderately correlated with CBF-LI in the ROIs of the MCAa and MCAp (Fig 7). Scatter plot graphs that show the correlation coefficient between CBF-LI and MEG-LI in ROIs of the MCAa and MCAp are presented in S1 Fig.

No significant correlation was recognized between MEG-LI and OEF-LI in any ROI.

### Differences in slow-wave distributions in the cerebral hemisphere in relation to the degree of vascular and metabolic impairment

Table 3 shows the results of statistical analysis of MEG for each ROI of the hemisphere related to the degree of vascular and metabolic impairments.

**Table 3:** Statistical analysis of MEG between Groups 1 and 2 for each ROI.

MEG	ROI	LI (mean ±SD)			M-W U	logistic regression		
		n = 37	Group 1 (n = 30)	Group 2 (n = 7)	p value	odds ratio [95%CI]		p value
0.3-4Hz	ACA	0.011 ±0.301	0.009 ±0.308	0.019 ±0.292	0.985	1.117	[0.070–17.708]	0.937
	MCAa	0.062 ±0.250	0.045 ±0.267	0.136 ±0.149	0.259	4.568	[0.152–137.715]	0.382
	MCAp	0.116 ±0.284	0.083 ±0.300	0.255 ±0.146	0.036*	7.362	[0.453–119.601]	0.160
	PCA	0.041 ±0.143	0.041 ±0.150	0.043 ±0.120	0.894	1.100	[0.003–371.752]	0.974
4-8Hz	ACA	0.014 ±0.195	0.012 ±0.209	0.023 ±0.132	0.925	1.364	[0.018–103.422]	0.888
	MCAa	0.107 ±0.291	0.061 ±0.302	0.303 ±0.113	0.029*	29.224	[0.849–1006.132]	0.062
	MCAp	0.120 ±0.298	0.067 ±0.296	0.351 ±0.191	0.009**	28.108	[1.227–644.056]	0.037*
	PCA	0.014 ±0.139	0.007 ±0.138	0.045 ±0.149	0.391	7.845	[0.017–3684.505]	0.512

Group 1: LI of CBF > -0.03 or LI of OEF < 0.01; Group 2: LI of CBF < -0.03 and LI of OEF > 0.01. LI: laterality index. M-W U: Mann-Whitney U test. ACA: anterior cerebral artery; MCAa: anterior middle cerebral artery; MCAp: posterior middle cerebral artery; PCA: posterior cerebral artery

*: *p<0.05*,

**: *p<0.01*

The Mann-Whitney U test showed statistically significant differences in 0.3- to 4-Hz wave laterality in the MCAp (*p < 0*.*05*) and 4- to 8-Hz wave laterality in the MCAa (*p < 0*.*05*) and MCAp (*p < 0*.*01*) between Group 1 and Group 2.

Univariate logistic regression analysis showed that 4- to 8-Hz wave laterality in the ROI of the MCAp was the sole statistically significant variable for the event of misery perfusion (Group 2) (odds ratio = 28.108 [95% CI, 1.227 to 644.056], *p* = 0.037).

**Fig 8:**
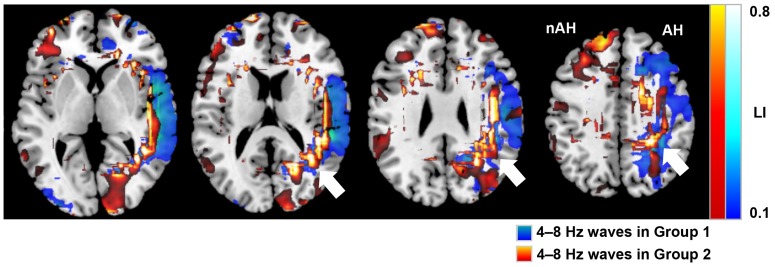
Imaging differences of theta wave (4–8 Hz) activity between the two groups of ischemic conditions. MRI overlaid with neuromagnetic slow waves showing differences in theta wave (4–8 Hz) distributions between the two groups of ischemic conditions: Group 1 (n = 30) and Group 2 (n = 7). Blue–green color indicates the distribution of 4- to 8-Hz waves in Group 1, whereas red–yellow color indicates the distribution of 4- to 8-Hz waves in Group 2. We found a significantly greater distribution of 4- to 8-Hz waves in the MCAp of the affected hemisphere in Group 2 compared with Group 1 (*p < 0*.*01*; white arrows). The images were anatomically standardized by SPM. AH: affected hemisphere, nAH: non-affected hemisphere.

Fig 8 illustrates differences in 4–8 Hz waves between Group 1 and Group 2. In Group 2, the 4- to 8-Hz wave distribution was predominantly from the posterior temporal to parietal regions (MCAp) of the affected hemisphere, in contrast to the distribution of Group 1.

## Discussion

### Non-arbitrary assessment of the cerebral ischemic area using automated ROI segmented with vascular territory

The characteristics of neuronal activity in various clinical situations must be taken into account when imaging neuromagnetic fields arising from the human brain. The ECD technique is a conventional source estimation method that is only feasible for evaluating neuronal activity recognized in a relatively localized area of the cerebrum as evoked activity or spike activity [18, 36–38]. Therefore, the ECD technique is unsuitable for analyzing spontaneous activity with multiple sources of an unknown number that generate various magnetic field patterns, as is the case in cerebral ischemia. As an alternative, sLORETA-qm has been employed as a source estimation method that does not require prior knowledge of the number of current sources [23]. This technique can provide information not only regarding localization, but also the absolute intensity of slow-wave activities in ischemic areas [24].

However, these existing methods are highly operator dependent because analysts subjectively select channels or subjectively decide the number of sources and source locations for the evaluation of cerebral slow-wave distribution in the ischemic brain. Moreover, they are not necessarily appropriate methods of investigation for patients with ischemic cerebrovascular disease, in whom evaluation of the cerebral vascular territory is preferable, because the calculated current intensity is expressed as the maximum value of the distribution. Therefore, in the present study, we optimized automated ROI constitution [33] for MEG quantitative imaging of patients with ischemic cerebrovascular disease by employing anatomical standardization, with the cerebral vascular territory currently used in SPECT imaging set as the ROI, which enables cerebral slow-wave distribution and intensity to be evaluated congruently with the cerebral vascular territory. We also applied the same ROI used in the MEG images to the ^15^O-PET images, enabling direct comparison between the two completely different imaging modalities of MEG and PET in a manner that was previously impossible.

### MEG distributions in the ischemic cerebral hemisphere caused by ICA occlusive lesions

A common belief is that any form of brain damage involving cortical gray matter and subcortical white matter will produce slow waves [39, 40]. Many previous studies detected slow waves in the cerebral hemisphere on the affected side in patients with occlusive disease of the ICA or MCA [20–22, 24, 41–43].

Many previous studies suggested that slow wave activity in the delta frequency range is highly typical in ischemic stroke [14, 16, 22, 24, 42–48] (Butz et al. [16], 0.5–3 Hz; Sakamoto et al. [22, 24], 0.3–4 Hz; Nuwer et al. [43], 0.1–4 Hz; Nagata et al. [44], 0.4–4 Hz; Nagata et al. [45], 2.0–3.8 Hz; Finnigan et al. [47], 1–4 Hz; Burghaus et al. [48], <1 Hz). Furthermore, delta activity has the strongest correlation with regional cerebral blood flow in patients with supratentorial and unilateral cerebral infarction [44–46]. In the present study, delta waves in the MCAp showed statistically significant laterality and were strongly correlated with CBF of the MCAa and MCAp, whereas delta waves in the MCAa did not show statistically significant laterality and were weakly correlated with CBF of the MCAa and MCAp. Two reasons for these results are possible.

The first possible reason is the issue of dysautoregulation caused by ICA stenosis. ICA stenosis or occlusion results in the loss of cerebral autoregulation in the parietal area, which is most distant from the main trunk of the occlusive ICA [49]. At the site of dysautoregulation, CBF promptly decreases with a reduction in cerebral perfusion pressure [5]. This drop in cerebral blood flow in the parietal lobe generates delta waves.

The second possible reason can also be explained by the characteristics of MEG sensor sensitivity to cerebral magnetic fields. MEG is more sensitive to superficial dorsolateral activity than to frontal or interhemispheric cerebral activity [20]. Consequently, MEG may pick up the signal from the temporo-parietal area alone, even if abnormal activity exists over a wider area of the ICA system.

Most previous studies have also presented theta waves in patients with cerebrovascular disease and suggested a correlation between theta activity and cerebral ischemia [20–22, 24, 42–45, 50] (Seki et al. [20], 6–8 Hz; Ohtomo et al. [21], 6–8 Hz; Sakamoto et al. [22, 24], 4–8 Hz; Nuwer et al. [43], 4.1–8 Hz; Nagata et al. [44], 4–7.6 Hz; Nagata et al. [45], 4–7.8 Hz). In the present study, theta waves displayed significant laterality in both the MCAa and MCAp regions. Theta waves were also moderately correlated with CBF in both the MCAa and MCAp regions, with no great difference in correlation coefficients between them (MCAa: MCAa *r =* 0.59, MCAp *r =* 0.54; MCAp: MCAa *r =* 0.60, MCAp *r =* 0.63). An increase in theta activity correlates with a decrease in blood flow in cerebral ischemia, although the degree of correlation is not as high as for delta activity, which was previously described [44].

However, two issues must be taken into account when considering the distribution of theta waves in the ischemic brain. The first is the association between age and theta waves. Theta waves are not uncommon in elderly people. Previous EEG studies have identified slow waves in elderly healthy volunteers over the age of 60 and reported that the incidence of theta rhythm increases with normal aging [51–53]. Theta waves may not be related to the underlying pathology of the ischemic brain. However, Seki et al. suggested that the presence of theta activity was significantly correlated with ICA occlusive lesions, regardless of the presence or size of infarct lesions [20]. They postulated that theta waves were strongly correlated with the lesion side, indicating the pathophysiological nature of this rhythm. Another study suggested that subclinical rhythmic electrographic discharges of adults, which are characterized by rhythmic, sharply contoured 5- to 7-Hz theta waves, are recognized in the parieto-occipital watershed area in older individuals and are postulated to be associated with cerebrovascular disease [50]. Peripheral circulatory insufficiency caused by cerebral arteriosclerosis in elderly people may cause theta wave generation.

The other problem is interhemispheric or transcallosal diaschisis, with slowing contralateral to the presumed side of the stroke. Some series of previous EEGs in stroke patients suggested increased numbers of theta waves bilaterally compared with normal subjects [12, 42, 54, 55]. The authors speculated that contralateral theta waves were probably caused by remote functional or metabolic effects on the normal hemisphere produced by the ischemic hemisphere. Our patients in the present study were also imaged for theta wave distributions in the parietal area that was contralateral to the affected ICA lesion (Fig 4, yellow arrows). The corpus callosum, which is the thickest commissural fiber in the human brain, the posterior part of which connects the bilateral parietal lobes, may play a crucial role for this phenomenon. This transcallosal diaschisis may have led to a reduction in statistical power because slow waves were evaluated by LI. However, we found a statistically significant laterality of theta waves in the territory of the MCA on the affected side. Significant augmentation of theta sources may be related to pathophysiological ischemic phenomena caused by the ICA occlusive lesion of the affected side.

### Theta-wave imaging potential for assessing the area of misery perfusion “ischemic penumbra”

Ischemic penumbra has been described as a region in which cerebral blood flow reduction has exceeded the threshold for failure of electrical function, but not of membrane function [1]. It is related to a dynamic process of impaired perfusion and metabolism that eventually propagates with time from the center of the area of ischemia to the neighboring tissue, and has recently been extended to characterize ischemically affected but still viable tissue with uncertain chances for infarction or recovery [42, 56]. This concept of ischemic penumbra entails the area of misery perfusion. Misery perfusion, a recoverable and treatable type of hemodynamic impairment, is evaluated by measures of hemodynamic status such as CBF and oxygen metabolism or cerebral perfusion reserve [57]. Previous PET and SPECT studies have demonstrated that misery perfusion is a risk factor for cerebral infarction [5, 7, 58]. For this reason, identification of the ischemic penumbra and the implementation of appropriate interventions before the development of cerebral infarction are of great importance in the treatment of cerebral stroke today.

Markedly decreased hemodynamic reserve without marked neurological impairment can occur in major cerebral arterial steno-occlusive disease. In such settings, the severity of cerebral ischemia does not indicate whether neural activity is well maintained or disturbed. Only minimal neurological symptoms develop, despite a marked decrease in hemodynamic reserve [49, 59]. However, clinical examination cannot determine whether or not the neuronal damage is reversible, and even DWI cannot necessarily detect irreversible tissue damage [48, 60]. The present study was also undertaken to examine slow neural activity in minor ischemic lesions resulting from major cerebral arterial occlusive disease of the ICA system.

When we divided theta wave distributions into two groups according to the severity of the ischemic brain and analyzed these distributions, a significant difference was evident, with theta waves distributed predominantly in the parietal lobe in the group with low cerebral vascular reserve (misery perfusion). The parietal watershed area, most distant from the main trunk of the ICA, appears vulnerable to a reduction in cerebral perfusion pressure and to have a tendency to develop cerebral infarction because of hemodynamic factors [49, 61]. Focal theta wave activities may be produced by this hemodynamic impairment that is responsible for the ICA occlusive lesion. Thus, theta wave distributions clearly imaged in the present study may identify an area of mild or subclinical ischemia in the ICA territories at risk for cerebral infarction (the so-called ischemic penumbra). This result suggests that MEG may be useful for detecting an ischemic area that is recoverable with appropriate treatment options before it manifests as a complete stroke. This has not been established in previous studies.

### Limitations of the present study

Some issues of the proposed imaging method require resolution. The present study did not include MEG and PET data from healthy volunteers. In particular, PET is an invasive examination that involves some degree of burden, due to the risks associated with radiation exposure and the need for arterial blood sampling. These risks prevented us from acquiring data from healthy people that could determine the cut-off value of normal limits. We therefore employed the presence of ipsilateral lesions that could be evaluated by LI as an enrollment criterion for patients in the present study. To enable evaluation of patients with bilateral lesions, future studies must also gather data from healthy volunteers.

The conductor model applied in the proposed method was spherical, whereas the actual brain is not completely spherical. Because voxels with spatially or locally maximum intensity at each time point were chosen in sLORETA-qm, the distributions of slow waves represent the gravity point of a large activated area that is estimated to be deeper than the true sources, as in the ECD method. The accuracy of estimated sources with MEG is influenced by the accuracy of the applied volume conductor. The use of more accurate models such as a realistic head shape model or a multisphere model would enable fitting of each sensor to the local curvature of the brain [62].

The number of subjects in the present study was small, including only 37 patients. In particular, when patients were divided into two groups according to their level of cerebral vascular reserve, with the cut-off value determined with reference to the median values and inter-quartile ranges of CBF and OEF, Group 2 contained only seven patients with severely impaired cerebral vascular reserve. This cannot be regarded as a sufficient number for statistical evaluation. However, statistical analysis demonstrated a correlation between temporo-parietal theta activity and a severe ischemic condition, a result that is not inconsistent with the findings of previous studies [20, 21]. Further studies with a larger number of patients are required.

## Conclusions

Frequency analysis of quantitatively imaged cerebral magnetic fields, based on sLORETA-qm using the automated ROI setting, enabled clear depiction of the distribution of spontaneous neuromagnetic activities in ischemic areas. Delta waves (0.3–4 Hz) were strongly correlated with a decrease in CBF, whereas theta waves (4–8 Hz) were distributed in the affected parietal area in association with a decrease in cerebral vascular reserve. Although further studies in a larger number of patients are warranted, the electrophysiological dynamics revealed by this non-invasive imaging technique demonstrate unique neural activity in the ischemic brain that is distributed in the impaired vascular metabolic territory, in which cerebral infarction has not yet been completed.

## Supporting Information

S1 Fig Scatter plot graph showing the correlation coefficient between CBF and MEG in each ROI. This graph shows the correlation coefficient between CBF-LI and MEG-LI in the ROI of the MCAa and MCAp, which corresponds to Fig 7 in this paper.
(PPTX)

S1 Table Correlation coefficient between MEG-LI and CBF-LI or OEF-LI in each ROI. Detailed results of the correlation coefficients between the MEG-LI and CBF-LI or OEF-LI in each ROI are shown in this table, which corresponds to Fig 7 in this paper.
(XLS)
