# Supplementary material for: MEG Frequency Analysis Depicts the Impaired Neurophysiological Condition of Ischemic Brain
Source: PLoS One. 2016 Dec 16;11(12):e0168588. doi: 10.1371/journal.pone.0168588 (PMC5161380; doi:10.1371/journal.pone.0168588)
Supplement: S1 Fig — This graph shows the correlation coefficient between CBF-LI and MEG-LI in the ROI of the MCAa and MCAp, which corresponds to Fig 7 in this paper. (PPTX) [file pone.0168588.s001.pptx]

## Slide 1
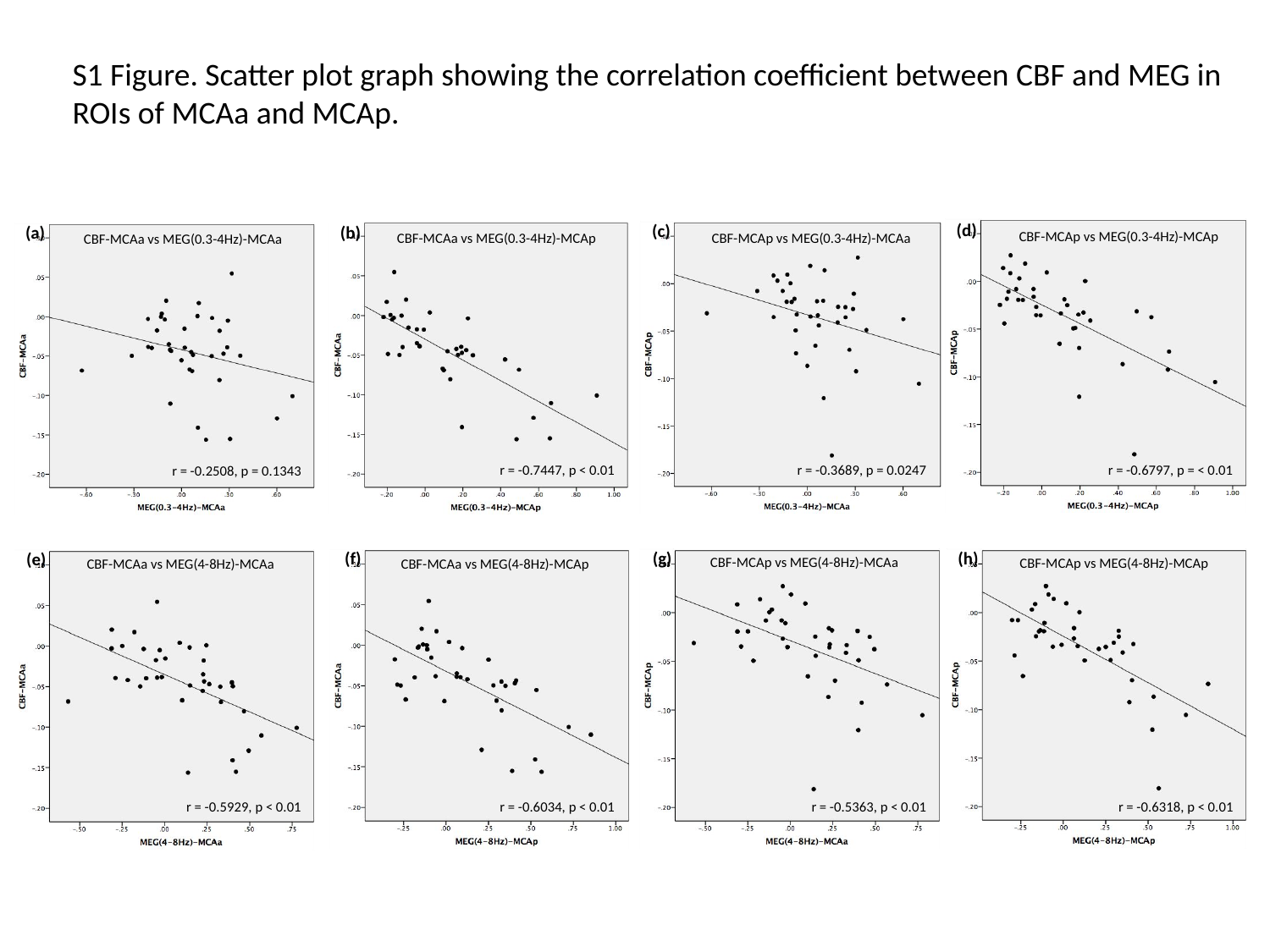

S1 Figure. Scatter plot graph showing the correlation coefficient between CBF and MEG in ROIs of MCAa and MCAp.
(d)
(c)
(a)
(b)
CBF-MCAp vs MEG(0.3-4Hz)-MCAp
CBF-MCAa vs MEG(0.3-4Hz)-MCAp
CBF-MCAp vs MEG(0.3-4Hz)-MCAa
CBF-MCAa vs MEG(0.3-4Hz)-MCAa
r = -0.7447, p < 0.01
r = -0.3689, p = 0.0247
r = -0.6797, p = < 0.01
r = -0.2508, p = 0.1343
(g)
(h)
(f)
(e)
CBF-MCAp vs MEG(4-8Hz)-MCAa
CBF-MCAp vs MEG(4-8Hz)-MCAp
CBF-MCAa vs MEG(4-8Hz)-MCAp
CBF-MCAa vs MEG(4-8Hz)-MCAa
r = -0.5929, p < 0.01
r = -0.6034, p < 0.01
r = -0.5363, p < 0.01
r = -0.6318, p < 0.01
